# Right ventricular function and dimensions in patients undergoing transcatheter aortic valve replacement assessed by three-dimensional echocardiography

**DOI:** 10.1007/s00392-024-02530-2

**Published:** 2024-09-05

**Authors:** Lukas Stolz, Simon Schmid, Julius Steffen, Philipp M. Doldi, Hans D. Theiss, Kornelia Löw, Magda Haum, Steffen Massberg, Jörg Hausleiter, Simon Deseive

**Affiliations:** 1 Medizinische Klinik und Poliklinik I, LMU Klinikum, LMU München, Marchioninistr. 15, 81377 Munich, Germany; 2 German Center for Cardiovascular Research (DZHK), Partner Site Munich Heart Alliance, Munich, Germany

**Keywords:** Three-dimensional echocardiography, Remodeling, Right ventricular function, Tricuspid regurgitation, TAVR, TAVI

Sirs,

Aortic stenosis (AS) is a major health burden leading to substantial morbidity and mortality. Transcatheter aortic valve replacement (TAVR) emerged as an important pillar in the treatment of severe AS. Within the past few years, right ventricular dysfunction (RVD) has been identified as an adverse prognostic marker in multiple cardiovascular disease entities [1]. Due to its complex anatomy and function, the right ventricle (RV) is difficult to characterize by means of two-dimensional echocardiography. Therefore, three-dimensional echo imaging (3DE) evolved as important diagnostic tool in the quantification of RV function and dimensions. Reduced 3D RV ejection fraction (RVEF^3D^ < 45%) was associated with lower postprocedural survival in patients undergoing edge-to-edge tricuspid valve repair [2, 3]. The aim of this study was to assess the impact of 3D RV dysfunction and dilation on outcomes in patients undergoing TAVR.

We included patients with available 3DE of the RV who underwent TAVR for severe AS from 2019 until 2021 at a high-volume heart valve center. 3DE included RV end-diastolic volume (RVEDVi^3D^), RV end-systolic volume (RVESVi^3D^, both indexed to the body surface area) and RV ejection fraction (RVEF^3D^). Depending on the pattern of RV dilation (RVEDVi > 87 ml/m^2^ for men and > 74 ml/m^2^ for women) and RV dysfunction (RVEF < 45%) based on current guideline recommendations [4], three groups of TAVR patients were defined: (1) normal RV function and dimensions (2) either RV dilation or dysfunction and (3) RV dilation and dysfunction. Study endpoints were two-year survival and improvement in heart failure symptoms as expressed by New York Heart Association (NYHA) functional class at latest available follow-up. The study adheres to the principles outlined in the declaration of Helsinki and was approved by the local ethics committee (EVERY-Valve registry, project number 19-840).

The study included 404 patients at a mean age of 81.6 ± 6.2 years (45.3% females). All patients suffered from severe AS as indicated by mean aortic valve pressure gradients of 36.7 ± 13.9 mmHg, a mean aortic valve opening area of 0.73 ± 0.19 cm^2^. Mean left ventricular function was 50.7 ± 9.5%. 3DE assessment of the RV revealed overall preserved RV dimensions (RVEDVi^3D^ 65.9 ± 21.3 ml/m^2^; RVESV^3D^ 38.8 ± 15.2 ml/m^2^) and mildly reduced RV function (RVEF^3D^ 41.5 ± 9.9%). Overall, 128 patients (31.7%) presented with normal RV function and dimensions, 210 patients (52.0%) with either RV dilation or dysfunction and 55 (16.3%) with RV dilation and dysfunction. 62.9% of patients presented with RV dysfunction and 21.8% with RV dilation.

**Fig. 1:**
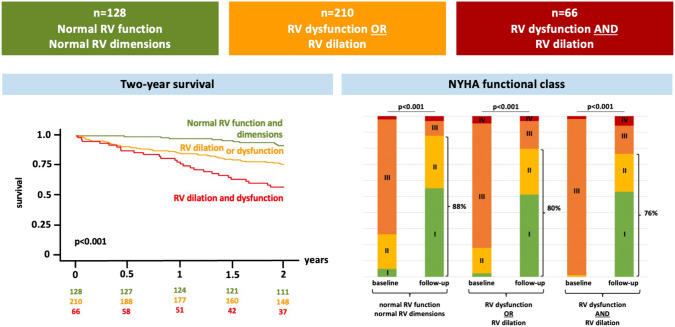
Right ventricular function and dimensions in TAVR patients assessed by three-dimensional echocardiography. *3D* three-dimensional; *NYHA* New York Heart Association functional class; *RV* right ventricular; *TAVR* transcatheter aortic valve replacement

Two-year survival status was available in 94.1% of patients. With increasing RV involvement, two-year survival rates differed by group assignment (normal RV function and dimensions: 92.0% vs. RV dysfunction or dilation 77.0% vs. RV dysfunction and dilation 58.6%, p < 0.001, Fig. 1). In a multivariable Cox regression model including all parameters showing statistical significance in a univariate analysis, RV dysfunction or dilation (HR 2.53; CI 0.96–6.63, p = 0.060) and RV dysfunction and dilation (HR 5.38; CI 1.97–14.64; p < 0.001) were independently associated with two-year all-cause mortality along with impaired renal function and increasing NYHA class. Of note, tricuspid regurgitation did not predict mortality in a multivariable analysis. NYHA functional class indicated more severe heart failure symptoms with progressive RV dilation and dysfunction at both, baseline, and follow-up (Fig. 1). Nevertheless, NYHA class significantly improved irrespective of RV size and function.

The present study reports outcomes of the largest TAVR cohort with available 3DE of the RV existing today. Using guideline recommended cut-offs for defining RV dilation and function, different patterns of RV pathologies were observed, yielding prognostic significance in terms of two-year survival. This emphasizes, that even though AS is a “left sided” pathology, right ventricular involvement in those patients occurs as a continuous spectrum which gradually impairs prognosis. 3DE is of special prognostic value in accurately quantifying the degree of RV involvement and might improve prognostic considerations prior to TAVR. The degree of RV damage in patients with AS might help optimizing treatment timing for TAVR. Even though patients with RV involvement presented with more severe heart failure symptoms, TAVR was associated with symptomatic improvement irrespective of RV function and dimensions. Whether there is a certain degree of RV dysfunction at which AS patients do no longer profit from treatment in terms of survival or symptomatic benefit cannot be answered in the setting of this retrospective study. Although the echocardiographic analyses were no subject to core laboratory supervision, the evaluations were performed by experienced echocardiographers according to recent imaging guidelines.

Right ventricular 3DE is of prognostic value in patients undergoing TAVR for severe AS. Since especially the presence of RV dilation and dysfunction at the same time is associated with worse survival prognosis, 3DE-derived RV function should be included into preprocedural patient evaluation.
